# Dietary intervention reprograms bone marrow cellular signaling in obese mice

**DOI:** 10.3389/fendo.2023.1171781

**Published:** 2023-07-10

**Authors:** Yuxuan Zheng, Jiren Yan, Xiaofu Zhang, Hailong Cui, Zhenyuan Wei, Xiaoying Li, Qiuyu Wang, Biao Zhong

**Affiliations:** ^1^ Institute of Metabolism and Integrative Biology, Fudan University, Shanghai, China; ^2^ Department of Orthopedic Surgery, and Shanghai Institute of Microsurgery on Extremities, Shanghai Sixth People’s Hospital Affifiliated to Shanghai Jiao Tong University School of Medicine, Shanghai, China; ^3^ Ministry of Education Key Laboratory of Metabolism and Molecular Medicine, Department of Endocrinology and Metabolism, Zhongshan Hospital, Fudan University, Shanghai, China; ^4^ Academy of Medical Sciences, Zhengzhou University, Zhengzhou, China; ^5^ First Affifiliated Hospital of Zhengzhou University, Zhengzhou University, Zhengzhou, China; ^6^ Department of Orthopaedics, Tongren Hospital, Shanghai Jiao Tong University School of Medicine, Shanghai, China

**Keywords:** obesity, bone formation, bone mass accrual, diet intervention, Wnt signaling pathway

## Abstract

**Objectives:**

The current study aimed to investigate the pathogenesis of obesity-induced impaired bone mass accrual and the impact of dietary intervention on bone density in the mouse model of obesity.

**Methods:**

Mice were fed with chow diet (CD) for 10 months, high-fat-diet (HFD) for 10 months, or HFD for 6 months then transferred to chow diet for 4 months (HFDt).

**Results:**

Weight loss and decreased intrahepatic lipid accumulation were observed in mice following dietary intervention. Additionally, HFD feeding induced bone mass accrual, while diet intervention restrained trabecular bone density. These changes were further reflected by increased osteogenesis and decreased adipogenesis in HFDt mice compared to HFD mice. Furthermore, HFD feeding decreased the activity of the Wingless-related integration site (Wnt)-β-Catenin signaling pathway, while the Wnt signaling was augmented by diet intervention in the HFDt group.

**Conclusions:**

Our findings suggest that a HFD inhibits bone formation and that dietary intervention reverses this inhibition. Furthermore, the dietary intervention was able to compensate for the suppressed increase in bone mass to a level comparable to that in the CD group. Our study suggests that targeting the Wnt signaling pathway may be a potential approach to treat obesity-induced impaired bone mass accrual.

## 1 Introduction

The global prevalence of obesity is estimated to reach 18% in men and 21% in women by 2025, representing a substantial health risk and economic burden (1). Obesity is commonly associated with dysregulated glucose, energy and fat metabolism, which can lead to an array of tissue-specific declines and dysfunctions (2, 3). Although current treatments for obesity often include bone mineral density decline as a side effect, this phenomenon is further exacerbated by the development of osteoporosis (OP) (4). Characterized by weakened bone microarchitecture and a decrease in bone density, OP is the most common bone disease worldwide, affecting an estimated 200 million people (5), and is a risk factor for various secondary health issues and mortality (6). Both obesity and OP have been found to have associations with nutrition, energy intake and sedentary lifestyles (7, 8).

Increasing evidence has shown that obesity, particularly due to excessive dietary fat intake, is associated with a heightened risk of fracture through disruption of bone remodeling and accelerated bone aging (9, 10). Studies have demonstrated a 33% body fat threshold at which visceral fat may produce a range of molecules with deleterious effects on the bone microenvironment (11, 12). Potential mechanisms underlying this phenomenon may include oxidative stress (13), elevated production of inflammatory cytokines (14), increased expression of peroxisome proliferator-activated receptor gamma (*PPARγ*) in bone marrow adipocytes (15), and disruption of Wnt signaling (16). The Wnt pathway is a key regulator of bone quantity and remodeling, largely due to its promotion of osteoblast differentiation and indirect control of osteoclastogenesis (17). The Wnt signaling pathway, comprising key molecules (Wnt3a, Wnt5a), has been identified as playing a pivotal role in bridging intrinsic processes associated with bone remodeling and energy metabolism, thus providing a promising avenue for the management of bone mass accrual (18).

Dietary interventions for weight loss are a well-established technique for managing obesity, with lifestyle modifications, such as dietary changes to decrease energy intake, being amongst the most widely investigated approaches (19). Thus, it is pertinent to ask whether the metabolic impairments in bone caused by a HFD can be reversed through a shift to a normal diet. Recent studies have exhibited a reversal of metabolic alterations, as well as a decrease in body weight, improved glucose tolerance, and decreased adiposity (20, 21), but little is known about the effects of changing diet on bone density. Scheller et al. demonstrated in a descriptive study that while a switch from a HFD to a CD could lead to weight loss, it could not fully prevent reduced bone formation. To better understand the effects of obesity-related reduced bone formation and the impact of a long-term dietary intervention on bone density, our study used mouse models of obesity and dietary restriction of fat components to induce weight loss.

## 2 Methods

### 2.1 Experimental animals

C57BL/6 male mice (6 weeks old) were obtained from the Laboratory Animal Center of Fudan University and housed in a specific pathogen-free environment (temperature: 23 ± 1°C; light-dark cycle: 12/12 hrs). Autoclaved food and water were provided ad libitum. Following a one-week acclimatization period, the animals were randomly divided into three groups: chow diet (CD; n=10), high-fat diet (HFD; n=12), and high-fat diet-transfer (HFDt; n=10). The CD group was fed a standard chow diet (Research Diets, D12450B, 10% cal% fat) for 10 months; the HFD group was fed a HFD (Research Diets, D12492, 60% cal% fat) for 10 months; and the HFDt group was fed a HFD for 6 months and then transferred to a chow diet for 4 months. HFDt group mice gradually increased body weight in the first 6 months, and then rapidly decreased to the same body weight as the CD group in the following 4 months. After 10 months of treatment, mice were fasted for 12 h and humanely euthanized under general anesthesia. To enrich the data on the relationship between male obesity and osteoporosis and reduce the confounding factors in the results, we used male mice for the model. All experimental protocols were approved by the Animal Care and Use Committee of Zhongshan Hospital, Fudan University.

### 2.2 Analysis of bone microstructure by micro-computed tomography

Following sacrifice, femurs were detached and fixed in 4% paraformaldehyde for subsequent micro-CT scanning and analysis. High-resolution imaging of the bone microstructure was carried out using a Skyscan 1176 system [software=Version 1.1 (build 6), Bruker, Kontich, Belgium] with a resolution of 8.96 microns. Trabecular bone mass was assessed by contouring a region 1.0 mm wide, positioned 500 microns from the proximal end of the distal femoral growth plate, and a threshold of 66–255 permille was applied. For the femoral cortical bone, a 500-micron-wide region was contoured starting 4.0 mm from the proximal end of the distal femoral growth plate, with a threshold of 114–255 permille. Three-dimensional reconstructions were created from the two-dimensional images acquired from the contoured regions.

### 2.3 Enzyme linked immunosorbent assay

Measurement of serum PINP in mice was conducted using the mouse procollagen I N-terminal propeptide (PINP) ELISA Kit (MU30602, Bioswamp) in accordance with the manufacturer’s instructions. Measurement of serum CTX-1 in mice was conducted using the mouse type I collagen C-terminal peptide (CTX-1) ELISA Kit (Jining Shiye, China) in accordance with the manufacturer’s instructions

### 2.4 Histology and immunostaining

Femurs and tibias were fixed in 4% paraformaldehyde for 48 h and incubated in 20% ethylenediaminetetraacetic acid (EDTA) solution for decalcification. Embedding in paraffin, dehydration, and cutting of 5 mm longitudinal sections were performed prior to staining with H&E, osteocalcin (OCN), and tartrate-resistant acid phosphatase (TRAP) (Sigma, Merck, Germany). After preparation of paraffin sections of the femur, they were first stained with hematoxylin staining solution, followed by eosin staining solution after dehydration with alcohol, and then dehydrated and sealed. The number of adipocytes per field of view was quantified using the Image J program. It is important to note that this method of fixing femoral adipocytes resulted in the degradation of a portion of the adipocytes and resulted in their inability to be identified. Immunohistochemistry following the IHC paraffin protocol (Abcam) with a OCN antibody (Proteintech, 23418-1-AP, 1:200) was performed and the proportion of positive cells in each field was quantified using the Image J program.

### 2.5 Quantitative RT-PCR analysis

Trizol reagent (Invitrogen) was used to extract total RNA from tibia, and cDNA was subsequently synthesized using a PrimeScript RT Reagent Kit (TaKaRa, Tokyo, Japan), following the manufacturer’s instructions. Quantitative real-time PCR was subsequently performed using SYBR Green Premix Ex Taq (Takara, Japan) and Light Cycler 480 (Roche, Switzerland), with the 2^−ΔΔCt^ method used for data analysis and GAPDH as an internal control for normalization. The sequences of oligonucleotides employed for RT-PCR are listed in **Table S1**.

### 2.6 Western blot analysis

Western blotting was employed to assess protein expression levels in bones. Equal protein concentrations were subjected to SDS-polyacrylamide gel electrophoresis (SDS-PAGE) and transferred onto PVDF membranes (Milipore, Darmstadt, Germany). Subsequently, the membranes were blocked with 5% skim milk-PBS-Tween 20 for 1 h at room temperature and incubated with specific antibodies to PPARγ (1:1,000, P37231, CST), Runx2 (1:1000, AF2593, Beyotime), Wnt5a (1:1000, AF8358, Beyotime), Wnt3a (1:1000, AF8352, Beyotime), β-catenin (1:1000, AF0066, Beyotime), GAPDH (1:1000, AB_2736879, Abclonal), and β-Actin (1:1000, AB_2768234, Abclonal) overnight at 4°C. The blots were then washed with TBS-Tween 20 (TBST) and incubated with horseradish peroxidase (HRP)-conjugated secondary antibodies (1:5000) for 1 h at room temperature. Lastly, the blots were washed again and incubated with enhanced chemilumescent (ECL) substrates (Bio-rad) for 1 min and the Image J software was applied to analyze the blots.

### 2.7 Bone marrow cellularity, isolation, and culture of bone marrow mesenchymal stem cells

Isolation of bone marrow stromal cells (BMSCs) was achieved through flushing with Dulbecco’s modified Eagle’s medium (DMEM) containing low glucose (Vitrocell, Brazil) supplemented with 10% fetal calf serum (Vitrocell, Brazil), 100 IU/ml sodium penicillin G (Sigma-Aldrich, USA), and 100 μg/ml streptomycin (Sigma-Aldrich, USA) at 37°C in a 5% CO2-95% humidity atmosphere. BMSCs were isolated based on their capacity to adhere to plastic surfaces in cell cultures using the aforementioned low-glucose medium. Cells of passages 3–10 were used for differentiation assays, which involved seeding BMSCs in six-well plates and treating them with an osteogenic medium composed of 50 μg/ml ascorbic acid, 5 mM β-glycerophosphate, and 100 nM dexamethasone (all from Sigma). After seven days of differentiation, the cells were washed with PBS, fixed in 4% paraformaldehyde (PFA) for 2 min, and assayed with ALP Staining Kit (Beyotime, C3206).

### 2.8 Serum and liver biochemical assays

Serum total triglyceride (TG), total cholesterol (TC), low density liptein cholesterol cholesterol (LDL-C), and high density liptein cholesterol cholesterol (HDL-C) levels were detected using commercial kits (Jiancheng, China). Liver TG and TC levels were detected using content assay kits (Applygen, China).

### 2.9 Statistical analysis

Statistical analyses were conducted using GraphPad 8.0. One-way ANOVA was employed to assess the impacts of CD, HFDt, and HFD. Subsequent to the ANOVA, Tukey’s multiple comparison test was utilized to further investigate the outcomes. When the homogeneity of variance assumption is violated for a one-way ANOVA, Welch’s ANOVA can be conducted instead, and Games-Howell’s multiple comparisons test was utilized to further investigate the outcomes.

We conducted a power analysis on the study model. Our effect size was 0.40, alpha error was 0.05, total sample size was 30, the number of groups was 3, The analysis method was one-way ANOVA, and the power obtained by G.power software was 0.44.

## 3 Results

### 3.1 Diet intervention reprograms whole body metabolism in mice

**Figure 1:**
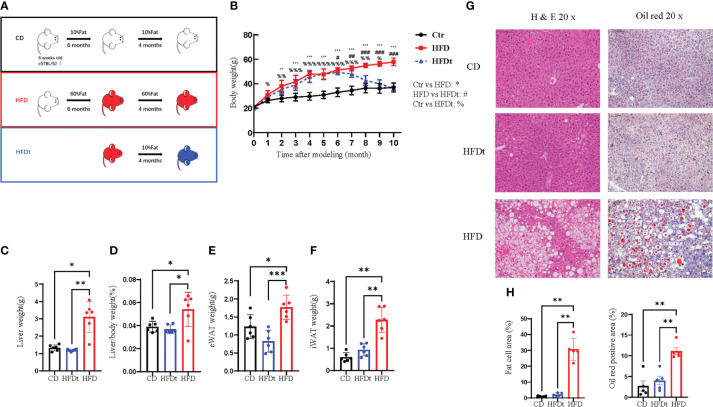
Diet intervention reprograms whole body metabolism in mice. **(A)** Experimental design: 6-week-old male C57BL/6 mice received a control diet (CD) (n = 10) or high-fat diet (HFD) (n = 20) ad libitum for 6 months. At the end of this period, half of the HFD group was switched to a chow diet for 4 months, referred to as the dietary intervention group (HFDt) (n = 10). **(B)** Body weight of mice after dietary intervention (n=10/group). **(C-F)** Liver weight, Liver weight/Body weight, Epididymal white adipose tissue (eWAT) weight, inguinal white adipose tissue (iWAT) weight (n = 6/group). **(G)** Histopathological analysis of H&E and oil red stained liver sections after CD, HFDt, or HFD treatment. **(H)** Quantification of H&E staining (left) and oil red staining (right) liver sections from the CD, HFDt, or HFD treated mice. All data shown were obtained from male animals. Significance was determined using one-way ANOVA or Welch’s ANOVA. *P < 0.05, **P < 0.01, ***P < 0.001, ^%^P < 0.05, ^%%^P < 0.01, ^%%%^P < 0.001, ^#^P < 0.05, ^##^P < 0.01, ^###^P < 0.001.

To examine whether dietary intervention could reverse the phenotype of long-term HFD induced OP in mice, we established HFD, HFDt, and CD groups to assess phenotypes of tibia, femur, and BMSCs (**Figure 1A**). In contrast to the HFD mice, the body weight of HFDt mice was significantly reduced (*P*<0.0001) after the 4 months of dietary intervention, and there was no significant difference (*P*>0.05) between the HFDt group and the CD group. At the end of the 10th month, the mean body weight ( ± standard deviation) of the HFD mice was 57.92 ± 2.99 g, and that of the HFDt and CD groups was 36.13± 3.48g and 36.91± 1.62g, respectively (**Figure 1B**). During the 4 months of diet intervention, caloric intake was lower in the HFDt group than in the CD group (**Figure S1A**). Liver weight (*P*<0.01), liver weight/body weight (*P*<0.05), eWAT (*P*<0.001), and iWAT (*P*<0.01) showed that HFD mice had a significantly greater tissue mass than HFDt and CD mice, indicating a normalization of their mass due to weight loss in HFDt mice. (**Figures 1C–F**). Serum TG levels were similar in all groups. Further examination of the levels of serum TC, HDL-C, and LDL-C revealed that the HFD group had higher levels than both the CD and HFDt groups (**Figures S1B-E**). Analysis of liver TG levels in mice exposed to a HFD demonstrated that TG levels were significantly higher in the HFD group than in both the CD (*P*<0.01) and HFDt (*P*<0.05) groups (**Figure S1G**). There were no differences in random blood glucose (RBG) between all groups (**Figure S1H**). Fasting blood glucose (FBG) levels were higher in HFDt mice than in CD mice, but significantly lower than in HFD mice (*P*<0.05), indicating that dietary intervention could improve blood glucose in HFD mice (**Figure S1I**). Liver tissues from HFD mice revealed an increased area of adipocytes and lipid droplets, suggesting that long-term HFD caused severe fatty liver, while dietary intervention ameliorated long-term HFD-induced fat accumulation (**Figures 1G, H**). Collectively, these results suggest that HFD feeding leads to obesity and metabolic disorders in mice, with dietary intervention offering a solution.

### 3.2 Diet intervention improves bone microarchitecture and promotes bone formation

**Figure 2:**
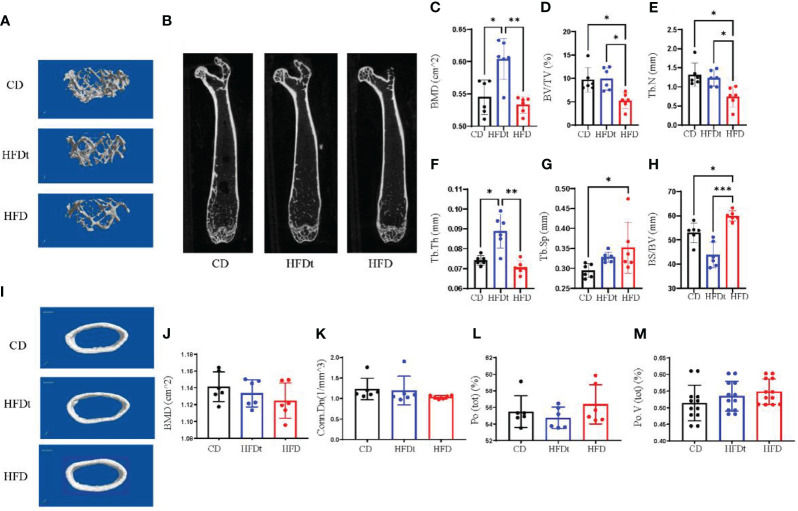
Diet intervention improves bone microarchitecture and promotes bone formation. **(A)** Micro-CT 3D reconstruction of representative images of tibial trabecular bone. **(B)** Sections of tibial trabecular bone from three groups. **(C-H)** Trabecular bone parameters at the distal femoral metaphysis, including BMD, BV/TV, Tb.N, Tb.Th, Tb.Sp and BS/BV after CD HFD or HFDt treatment (n = 6). **(I)** Micro-CT 3D reconstruction of representative images of tibial cortical bone. **(J-M)** Cortical bone parameters of the distal metaphysis of the femur were measured, including BMD, Conn.Dn, Ct.Po, Po.V (tot) after CD, HFD or HFDt treatment (n = 6). BMD, (Bone Mineral Density); BV/TV, trabecular bone volume per tissue volume; Tb. N, trabecular number; Tb.Th, trabecular thickness; Tb.Sp, trabecular separation; BS/BV, trabecular surface area per bone volume; Conn.Dn, connectivity density; Po (tot), Ct.Po, cortical porosity; Po.V (tot), total volume of pore space. Significance was determined using one-way ANOVA or Welch’s ANOVA. *P < 0.05, **P < 0.01.

To further explore whether dietary intervention improved bone microarchitecture in HFD mice, we performed micro-CT in three groups of mice. Analysis of micro-CT scans in three groups of mice indicated that femur microarchitecture was detrimentally impacted by HFD, with loose trabeculae and disordered structure (**Figures 2A, B, I**). In contrast, bone microarchitecture was maintained in the diet intervention group, with regular and closely arranged trabecular bone structure resembling that of the control group. This demonstrated significant improvement of bone microarchitecture in HFD mice post-dietary intervention. Subsequent trabecular bone parameters analysis with three software sets revealed that BMD (*P*<0.001), BV/TV (*P*<0.05), Tb.N (*P*<0.05), Tb.Th (*P*<0.01) and BS/BV (*P*<0.001) were significantly improved in the HFDt group compared to the HFD group, with most indexes similar or even better than those in the control group (P < 0.05). Our findings suggest that long-term HFD-induced disruption of bone microarchitecture in mice is evident, and can be effectively ameliorated by dietary intervention (**Figures 2C–H**). In the analysis of cortical bone, no significant difference was found between the HFDt group and CD group (*P*>0.05) (**Figures 2J–M**). The results of cancellous and cortical bone analysis further demonstrate that most parameters of the HFDt mice tibia were fully restored following dietary intervention.

### 3.3 Dietary intervention promotes bone formation by enhancing osteoblast activity

**Figure 3:**
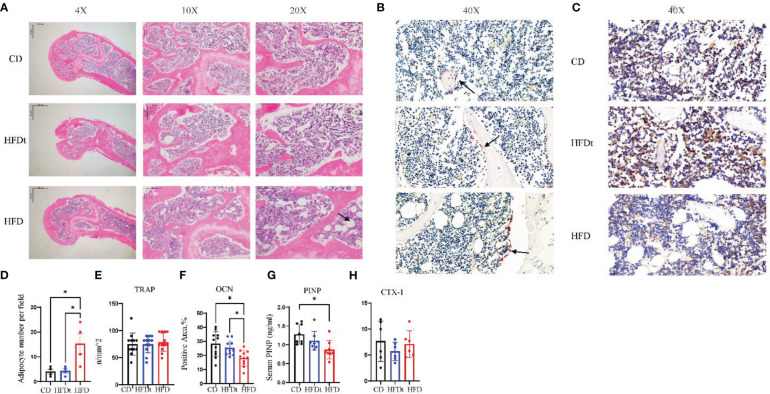
Dietary intervention promotes bone formation by enhancing osteoblast activity. **(A)** Representative images of femoral bone sections stained with H & E after CD, HFDt, or HFD treatment (Arrow indicates adipocytes). **(B)** Representative images of IHC staining for detecting the tartrate-resistant acid phosphatase (TRAP) expression, which reflects the osteoclast activity (Arrow indicates osteoclast). **(C)** Representative images of IHC staining for detecting the osteocalcin (OCN) expression, which reflects the osteoblast activity. **(D)** Analysis of adipocyte per field. **(E)** Analysis of trap-positive cell number per bone surface. **(F)** Analysis of Ocn-positive cell number per bone surface; **(G)** Levels of serum marker of bone formation. **(H)** Levels of serum marker of bone resorption. Significance was determined using one-way ANOVA or Welch’s ANOVA. *P < 0.05.

In the bone marrow microenvironment, the dynamic balance of adipogenesis and osteogenesis, and the number of osteoblasts and osteoclasts all have a major impact on bone microarchitecture. The femurs of three groups of mice were stained to identify adipose, OCN and TRAP in order to evaluate the effect of the dynamic balance of adipogenesis and osteogenesis on bone microarchitecture. Results indicated that long-term HFD had significantly increased number of adipocytes per field in the distal femur, compared to CD (*P*<0.05) and HFDt (*P*<0.05) mice, suggesting that dietary intervention ameliorated the accumulation of adipocytes caused by long-term HFD (**Figures 3A, D**). We further examined the osteoblast and osteoclast biomarkers OCN and TRAP (**Figures 3B, C**). TRAP staining of femur revealed no difference (*P*>0.05) in the number of osteoclasts between the three groups of mice (**Figure 3E**). However, OCN exhibited significant variances between the three groups. OCN content was significantly lower in HFD mice than in CD (*P*<0.05) and HFDt (*P*<0.05) mice (**Figure 3F**). Serum PINP levels in HFD mice were significantly lower than in CD mice, whereas those in the HFDt group returned to normal (**Figure 3G**). There was no significant difference in serum CTX-1 levels between the three groups (*P*>0.05) (**Figure 3H**). These results suggest that HFD feeding perturbs the equilibrium between osteogenic and adipogenic differentiation in mouse bone marrow, which leads to reduced bone formation. Dietary intervention, however, appears to restore the balance of osteogenic and adipogenic processes in mouse bone marrow, thus restoring its osteogenic potential.

### 3.4 Dietary intervention restores adipogenic and osteogenic balance in tibia and BMSCs

**Figure 4:**
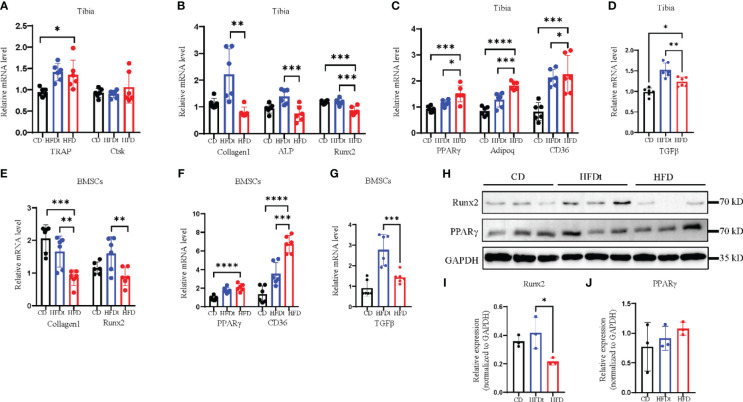
Dietary intervention restores adipogenic and osteogenic balance in tibia and BMSCs. **(A)** mRNA levels of osteoclastic genes in BMCs of CD, HFDt, or HFD mice (n = 6). **(B)** mRNA levels of osteoblastic genes in BMCs of CD, HFDt, or HFD mice (n = 6). **(C)** mRNA levels of adipogenic genes in BMCs of CD, HFDt, or HFD mice (n = 6). **(D)** mRNA levels of inflammatory genes in BMCs of CD, HFDt, or HFD mice (n = 6). **(E)** Immunoblot of Runx2, PPARγ protein expression in the bone of CD, HFDt, and HFD mice: each lane contains samples pooled from three mice, GAPDH is shown as a loading control. **(F, G)** The relative protein expression levels of Runx2, PPARγ. **(H)** mRNA levels of osteoblastic genes in BMSCs of CD, HFDt, or HFD mice (n = 6). **(I)** mRNA levels of adipogenic genes in BMSCs of CD, HFDt, or HFD mice (n = 6). **(J)** mRNA levels of inflammatory genes in BMSCs of CD, HFDt, or HFD mice (n = 6). Significance was determined using one-way ANOVA or Welch’s ANOVA. *P < 0.05, **P < 0.01, ***P < 0.001; ****P < 0.0001.

Analysis of mRNA expression in the tibia of mice was conducted to explore the metabolic mechanisms of dietary intervention on bone homeostasis. Relative expression of osteoclastic genes, *TRAP* and Cathepsin K (*Ctsk*), did not differ between HFD and HFDt mice (*P*>0.05) (**Figure 4A**). Conversely, relative expression of osteogenic genes, Collagen type I A I (*Col1a1*), alkaline phosphatase (*ALP*), and Runt-related transcription factor 2 (*Runx2*), were decreased in BMCs of HFD mice (**Figure 4B**). Moreover, relative expression of adipogenic genes, *PPARy*, adiponectin (*Adipoq*), and cluster of differentiation 36 (*CD36*), were increased in BMSCs of HFD mice (**Figure 4C**). Notably, relative expression of the inflammatory gene transforming growth factor beta-1 (*TGFβ*) was significantly increased in BMCs of HFDt mice compared to CD (*P*<0.0001) and HFD (*P*<0.0001) mice (**Figure 4D**). Further, the protein levels of *Runx2* and *PPARγ* in the tibia of mice were examined. *Runx2* protein expression was significantly higher (*P*<0.05) in HFDt mice than in HFD mice, while *PPARγ* protein expression was higher in HFD mice than in the other two groups (**Figures 4H–J**). The osteogenic and adipogenic balance of tibia is regulated by BMSCs, we further analyzed the mRNA expression in BMSCs. Results in mRNA expression in BMSCs revealed that, following HFD exposure, the relative expression of osteogenic genes (*Col1a1* and *Runx2*) was decreased (**Figure 4E**), while the relative expression of adipogenic genes (*CD36*, *PPARγ*) was increased (**Figure 4F**). Notably, the relative expression of the inflammatory gene (*TGFβ*) was significantly increased in BMSCs of HFD mice compared to CD (*P*<0.01) and HFD (*P*<0.05) mice (**Figure 4G**). These results indicate that HFD disrupts the balance of osteogenic and adipogenic differentiation in tibia and blunts bone formation, leading to bone loss. However, following dietary intervention, the balance of osteogenic and adipogenic differentiation in tibia was restored, which in turn restored the osteogenic potential. Furthermore, the results of BMSCs demonstrate the underlying mechanism by which dietary intervention ameliorates HFD-induced bone loss: dietary intervention could promote the differentiation of BMSCs toward the osteogenic direction, and inhibit the adipogenic capacity of BMSCs.

### 3.5 Dietary intervention improves local Wnt signaling pathway

**Figure 5:**
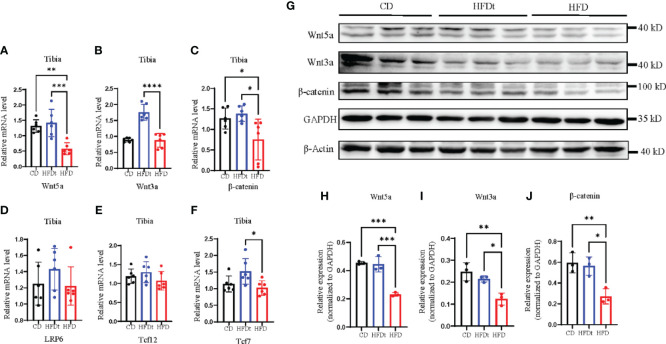
Dietary intervention improves local Wnt signaling pathway. **(A-F)** Tibia mRNA expression of the Wnt signaling molecules. **(G-J)** Immunoblot of Wnt signaling pathway protein expression in the bone of CD, HFDt, and HFD mice: each lane contains samples pooled from three mice, GAPDH is shown as a loading control. Significance was determined using one-way ANOVA or Welch’s ANOVA. *P < 0.05, **P < 0.01, ***P < 0.001; ****P < 0.0001.

Previous studies have indicated that HFD leads to bone loss and consumption may be associated with reduced activity of Wnt signaling pathways (22). Subsequent to these findings, our research evaluated the skeletal expression of both canonical and noncanonical Wnt signaling pathways in different groups of mice. mRNA and protein expression levels of *Wnt5a*, *Wnt3a*, β-catenin, and nuclear effectors *Tcf7l2* and *Tcf7* of the Wnt signaling pathway, as well as *LRP6*, were measured. Results showed that HFD feeding decreased skeletal *Wnt5a*, *Wnt3a*, and β-catenin mRNA expression and protein expression, compared to the control group (**Figures 5A–J**). However, in HFDt mice, Wnt signaling was augmented by dietary intervention, as indicated by increased expression of downstream signaling molecules.

## 4 Discussion

The global prevalence of obesity is on the rise, associated with a variety of metabolic disorders and complications, among which OP is particularly salient (23). Non-invasive dietary interventions may offer potential for ameliorating the effects of obesity on health. Despite the number of evidence demonstrating the deleterious effects of obesity on bone health, very few studies have comprehensively explored the implications of dietary interventions for obesity-induced OP (24, 25). The present study revealed that 10 months of HFD consumption led to increased body weight, liver weight, femoral trabecular bone loss, and dyslipidemia in mice. Subsequent 4 months of switching from HFD to a regular diet caused a notable improvement in bone microarchitecture and bone formation. The molecular mechanisms of HFD-induced impaired bone mass accrual and its reversal by diet intervention were further explored. It was observed that HFD caused downregulation of osteogenic genes (*Col1a1* and *Runx2*) and upregulation of adipogenesis genes (*CD36* and *PPARy*) as well as downregulation of genes and proteins in the Wnt/β-catenin signaling pathway. Remarkably, diet intervention reversed these implications of HFD on bone, and restoration of local Wnt/β-catenin signaling may explain the potential mechanism. The findings of this study demonstrate the efficacy of diet intervention in regulating obesity-induced osteoporosis and suggest that it may serve as a promising non-invasive approach to ameliorating metabolic disorders associated with obesity.

Evidence has suggested a link between obesity and OP (26), with aged populations being particularly susceptible to the simultaneous presence of both syndromes (27). Despite numerous attempts to control obesity through pharmacological treatments, several initially approved anti-obesity drugs have been withdrawn due to serious adverse effects, including bone loss (28, 29). In light of this, it is essential to determine the relationship and underlying mechanisms between obesity and OP. This study assesses the impact of HFD on bone formation in obesity mice, demonstrating a decrease in the transcription factor Runx2, which plays a fundamental role in osteogenesis. Moreover, HFD was found to have a suppressive effect on *Runx2* at the transcriptional level in tibia and BMSCs, as well as at the protein level in mice tibia. Furthermore, HFD increased the expression of *PPARγ*, a marker of adipogenesis, suggesting a role of HFD in impairing bone formation and enhancing bone marrow adipogenesis. These findings provide insight into the potential effects of obesity on bone health, and the involvement of the Wnt signaling pathway (30). It is worth noting that some indexes of the HFDt group were higher than those of the CD group. The reason for this phenotype is unknown, but we analyzed that HFDt may activate inflammatory path-related genes such as TGF-β, which can play a synergistic role with Runx2 to promote bone mass increase (31).

Previous research has demonstrated that obesity can lead to increased differentiation of BMSCs into adipocytes and reduced differentiation of BMSCs into osteoblasts (32). This observation has been attributed to the attenuation of the Wnt signaling pathway, which is a crucial regulator of bone formation, affecting the formation and function of osteoblasts, adipocytes, and osteoclasts (16, 33, 34). The canonical Wnt pathway is activated by ligands such as Wnt1 and Wnt3a and is mediated by β-catenin, which, upon activation, can translocate to the nucleus to induce the expression of target genes (16, 33, 34). Wnt5a, a noncanonical Wnt ligand, has been observed to activate both the canonical and noncanonical pathways. It does this by binding to canonical Wnt and modulating Wnt–β-catenin signaling in osteoblasts and certain stromal cell lines, as well as binding to receptor tyrosine kinase-like orphan receptor 1/2 (Ror1/2) to stimulate cell migration and polarization (35). In the Wnt signaling cascade, activated Wnt blocks glycogen synthase kinase 3 (GSK3)-catalyzed phosphorylation of β-catenin (36), leading to the subsequent translocation of unphosphorylated β-catenin into the nucleus and up-regulation of Runx2 expression (37). In the current study, we observed a downregulation of the canonical Wnt pathway components *Wnt3a*, *p-GSK*, and β-catenin in the skeleton of mice on a HFD, with a subsequent restoration of the canonical Wnt/β-catenin pathway following diet intervention (38). Wnt5a activates both the canonical and non-canonical Wnt pathways and has been observed to suppress adipogenesis, thereby promoting the differentiation of BMSCs into osteoblast lineage cells (39, 40). Further, we observed that Wnt5a suppressed transactivation of *PPARγ* and induced the expression of *Runx2*, leading to the promotion of osteogenesis (41). Interestingly, we also observed decreased Wnt5a signaling in the HFD group and a regained activity in the HFDt group. Our findings are consistent with previous work that revealed an increase in bone marrow adiposity and adipogenesis genes, as well as decreased bone density and osteogenesis genes, in HFD mice compared to HFDt and CD mice.

So far, lifestyle changes are considered the mainstay of the management of obesity. Dietary restriction has been shown to reduce diet-induced obesity and diabetes (42), reduced adipose tissue inflammation (43), intrahepatic lipid accumulation (44), and improved behavioral impairments (20). To our knowledge, little is known whether diet-induced weight loss may improve obesity-induced impaired bone mass accrual. As expected, weight loss reduced bone weight gain, liver weight, intrahepatic lipid accumulation, hyperlipidemia, glucose tolerance, which corresponds with previous studies. In support of this, our study showed that a 4-month dietary intervention reversed the impairments of HFD-induced bone density. These results provide further understanding of the relationship between diet-induced weight loss and bone health. Scheller et al. previously studied the effects of HFD and weight loss on male C57BJ/L mice, with duration of 12, 16, or 20 weeks and a group of mice fed HFD for 12 weeks and then on CD for 8 weeks to mimic weight loss. Contrary to results in our study, no statistically significant differences in femur trabecular morphology were found between the weight loss group and HFD group. We designed a longer duration of diet intervention, 16 weeks, and observed improved femur bone microarchitecture in the HFDt group. This difference may be attributed to the duration of weight loss, since Scheller’s study was limited to 8 weeks of diet intervention. Although no statistically significant changes were observed, a tendency of recovery was still present in Scheller’s work.

In this study, we demonstrate a link between obesity and bone formation, characterized by an increase in adipogenesis and a decrease in osteogenesis. Furthermore, these changes in bone architecture were reversed by dietary intervention, highlighting the potential of weight loss as a therapeutic strategy. Additionally, our findings suggest that obesity-induced impaired bone mass accrual may be due to the suppression of the Wnt signaling pathway, and that dietary intervention may restore its activity. The results of this study provide a basis for further exploration of the mechanisms that underlie obesity-induced impared bone mass accrual, and future research should focus on interventions that augment the Wnt signaling pathway to prevent or ameliorate this condition.

## Data availability statement

The datasets presented in this study can be found in online repositories. The names of the repository/repositories and accession number(s) can be found within the article/**Supplementary Material**.

## Ethics statement

The animal study was reviewed and approved by Animal Care Committee of Zhongshan Hospital, Fudan University.

## Author contributions

YZ, JY, XZ: Methodology, investigation, data analysis, writing—original draft. HC, ZW: Data analysis, conceptualization. XL: Funding acquisition. QW: Conceptualization, supervision. BZ: Methodology, resources, supervision. All authors contributed to the article and approved the submitted version.
