# Supplementary material for: Dietary intervention reprograms bone marrow cellular signaling in obese mice
Source: Front Endocrinol (Lausanne). 2023 Jul 10;14:1171781. doi: 10.3389/fendo.2023.1171781 (PMC10390309; doi:10.3389/fendo.2023.1171781)
Supplement: Supplementary file 1 [file DataSheet_1.docx]

Supplementary Material

**Dietary Intervention Reprograms Bone Density in Obese Mice**

**Yuxuan Zheng^2^**^†^**, Jiren Yan^1^**^†^**, Xiaofu Zhang^3^**^†^**, Hailong Cui^4 5^, Zhenyuan Wei^6^, Xiaoying Li^2 3^, Qiuyu Wang^3*^&Biao Zhong^1*^**

†These authors contributed equally to this work and share first authorship

^1^Department of Orthopedic Surgery, and Shanghai Institute of Microsurgery on Extremities, Shanghai Sixth People's Hospital Affiliated to Shanghai Jiao Tong University School of Medicine

^2^Institute of Metabolism and Integrative Biology, Fudan University, Shanghai, China

^3^ Ministry of Education Key Laboratory of Metabolism and Molecular Medicine, Department of Endocrinology and Metabolism, Zhongshan Hospital, Fudan University, Shanghai, China

^4^Academy of Medical Sciences, Zhengzhou University, Zhengzhou, China

^5^First Affiliated Hospital of Zhengzhou University, Zhengzhou, China

^6^Department of orthopaedics, Tongren Hospital, Shanghai Jiao Tong University School of Medicine, Shanghai, China

**Correspondence:**

Biao Zhong

[biao.zhong@sjtu.edu.cn](mailto:biao.zhong@sjtu.edu.cn)

# Supplementary Figures and Tables

## Supplementary Figures


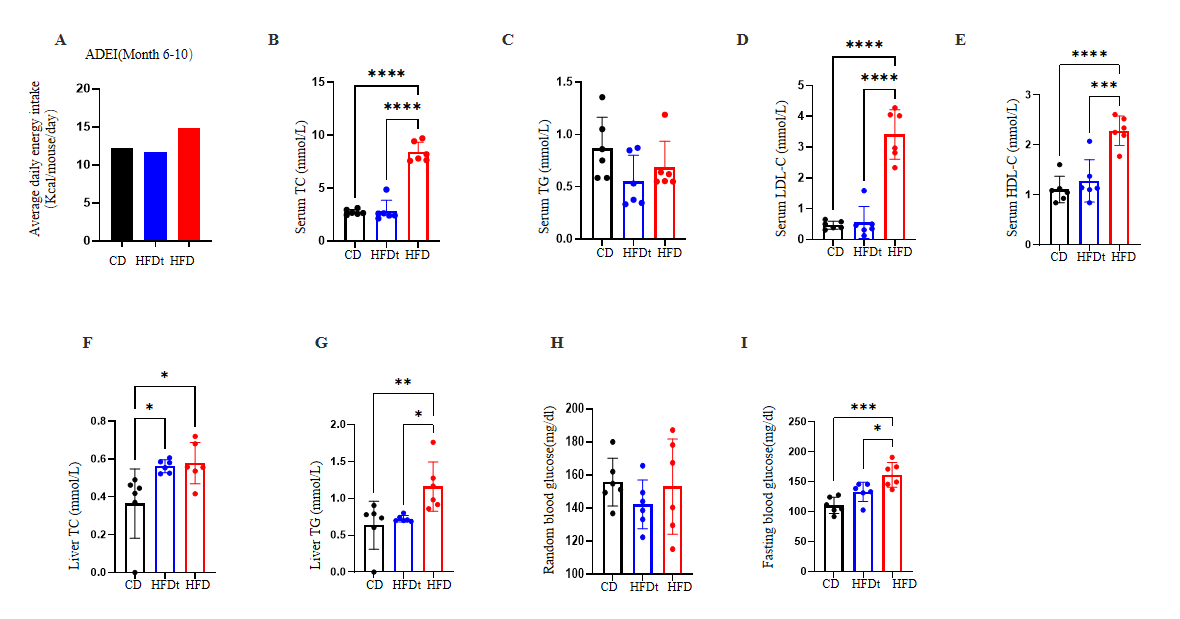


**Supplementary Figure 1**, related to Figure 1. Serum and liver metabolic indexes and blood glucose levels of the three groups of mice

**(A)** The average daily energy intake of three groups of mice during 4 months of diet intervention. **(B-E)** Total cholesterol (TC), Total Triacylglycerol (TG), high-density lipoprotein (HDL-C), and low-density lipoprotein (LDL-C) plasma levels in CD mice, HFDt mice, or HFD mice (n = 6/group). **(F-G)** Total cholesterol (TC), Total Triacylglycerol (TG) liver levels in the liver of CD mice, HFDt mice, or HFD mice (n = 6/group). **(H-I)** Random blood glucose and fasting blood glucose of the two groups (n = 6/group). Significance was determined using one-way ANOVA or Welch's ANOVA. **P* < 0.05, ***P* < 0.01, ****P* < 0.001; *****P* < 0.0001.

# 1.2 Supplementary Tables

| Target gene | Forward (5’ to 3’) | Reverse (5’ to 3’) |
| --- | --- | --- |
| β-actin | AGATTACTGCTCTGGCTCCTAGC | ACTCATCGTACTCCTGCTTGCT |
| Col1a1 | CTGGCGGTTCAGGTCCAAT | TTCCAGGCAATCCACGAGC |
| Runx2 | GGTACTTCGTCAGCATCCTATCAG | GCTTCCGTCAGCGTCAACAC |
| Pparγ | CACTCGCATTCCTTTGACATC | CGCACTTTGGTATTCTTGGAG |
| Adipoq | ATCTGGAGGTGGGAGACCAA | GGGCTATGGGTAGTTGCAGT |
| CD36 | GAGCA ACTGG TGGAT GGTTT | GCAGA ATCAA GGGAG AGCAC |
| TRAP | CAGCAGCTCCCTAGAAGATGG | CTGGAACCTCTTGTCGCTGG |
| Ctsk | GAAGAAGACTCACCAGAAGCAG | TCCAGGTTATGGGCAGAGATT |
| Alp | AACCC AGACA CAAGC ATTCC | GCCTT TGAGG TTTTT GGTCA |
| Tgf-β | CTCCCGTGGCTTCTAGTGC | GCCTTAGTTTGGACAGGATCTG |
| Wnt5a | CAACTGGCAGGACTTTCTCAA | CATCTCCGATGCCGGAACT |
| Wnt3a | CTCCTCTCGGATACCTCTTAGTG | GCATGATCTCCACGTAGTTCCTG |
| Lrp6 | TTGTTGCTTTATGCAAACAGACG | GTTCGTTTAATGGCTTCTTCGC |
| Catenin | ATGGAGCCGGACAGAAAAGC | CTTGCCACTCAGGGAAGGA |
| Tcf7 | ACCCTCCAGAATCCACAGATAC | ATGTTGCCTCCTCCTGAGTTAG |
| Tcf7l2 | GGAACTAGTCTTCTCTCAGCCAA | CGTCATCGGATTTGATCTCA |

**Supplementary Table 1|** Sequences of primers used for real-time quantitative PCR

|  | CD (mean±SD) | HFDt (mean±SD) | HFD (mean±SD) | CD vs HFDt 95.00% CI of diff | CD vs HFDt ES | CD vs HFD 95.00% CI of diff | CD vs HFD ES | HFDt vs HFD 95.00% CI of diff | HFDt vs. HFD ES |
| --- | --- | --- | --- | --- | --- | --- | --- | --- | --- |
| Body weight (g) | 36.91±3.664 | 36.13±1.709 | 57.92±3.157 | -22.68 to -4.976 | 0.14 | -23.93 to -1.533 | -0.95 | -10.10 to 12.30 | -0.97 |
| liver weight (g) | 1.328±0.1734 | 1.193±0.06772 | 3.107±0.8894 | -0.6517 to 0.9217 | 0.46 | -2.565 to -0.9916 | -0.81 | -2.700 to -1.127 | -0.84 |
| liver/body weight | 0.03903±0.004665 | 0.03689±0.003693 | 0.05406±0.01474 | -0.01161 to 0.01591 | 0.25 | -0.02879 to -0.001269 | -0.57 | -0.03094 to -0.003416 | -0.62 |
| eWAT (g) | 1.23±0.3314 | 0.8283±0.3071 | 1.767±0.3367 | -0.08625 to 0.8896 | 0.53 | -1.025 to -0.04875 | -0.63 | -1.426 to -0.4504 | -0.82 |
| iWAT (g) | 0.5983±0.1985 | 0.9283±0.2767 | 2.277±0.5611 | -0.8983 to 0.2383 | -0.57 | -2.247 to -1.110 | -0.89 | -1.917 to -0.7800 | -0.84 |
| Fat cell area (%) | 1.087±0.3868 | 2.143±1.012 | 30.7±7.033 | -7.989 to 5.876 | -0.57 | -36.55 to -22.68 | -0.95 | -35.49 to -21.62 | -0.94 |
| oil red positive area (%) | 2.688±2.763 | 4.008±2.306 | 11.15±1.887 | -5.279 to 2.639 | -0.25 | -12.42 to -4.499 | -0.87 | -11.10 to -3.179 | -0.86 |
| BMD (cm^2) | 0.545±0.02667 | 0.604±0.03189 | 0.5332±0.01262 | -0.09662 to -0.02138 | -0.71 | -0.02579 to 0.04945 | 0.27 | 0.03321 to 0.1085 | 0.83 |
| BV/TV (%) | 9.708±2.641 | 9.919±2.397 | 5.233±1.698 | -3.631 to 3.209 | -0.04 | 1.055 to 7.895 | 0.71 | 1.266 to 8.106 | 0.75 |
| Tb.N (mm) | 0.545±0.02667 | 0.604±0.03189 | 0.5332±0.01262 | -0.3051 to 0.4758 | -0.71 | 0.1814 to 0.9623 | 0.27 | 0.09606 to 0.8769 | 0.82 |
| Tb.Th (mm) | 0.07417±0.002401 | 0.08883±0.008448 | 0.07056±0.003324 | -0.09662 to -0.02138 | -0.76 | -0.02579 to 0.04945 | 0.53 | 0.03321 to 0.1085 | 0.82 |
| Tb.sp (mm) | 0.2942±0.01734 | 0.328±0.01287 | 0.352±0.06381 | -0.09216 to 0.02449 | -0.74 | -0.1162 to 0.0004937 | -0.53 | -0.08233 to 0.03433 | -0.25 |
| BS/BV (mm) | 53.04±4.049 | 53.04±5.27 | 59.95±2.076 | 3.165 to 15.22 | 0.70 | -12.94 to -0.8801 | -0.73 | -22.13 to -10.07 | -0.90 |
| Cortical BMD (cm^2) | 1.141±0.01769 | 1.134±0.01619 | 1.125±0.02094 | -0.01974 to 0.03539 | 0.20 | -0.01095 to 0.04418 | 0.38 | -0.01878 to 0.03636 | 0.23 |
| Conn.Dn (1/mm^3) | 1.232±0.264 | 1.194±0.3512 | 1.031±0.04103 | -0.3433 to 0.4208 | 0.06 | -0.1811 to 0.5830 | 0.47 | -0.2199 to 0.5443 | 0.31 |
| Po(tot) (%) | 55.5±1.9 | 54.76±1.287 | 56.38±2.367 | -2.112 to 3.597 | 0.22 | -3.736 to 1.973 | -0.20 | -4.479 to 1.230 | -0.39 |
| Po.V(tot) (mm^3) (%) | 0.5139±0.054 | 0.535±0.04503 | 0.5478±0.03811 | -0.06737 to 0.02514 | -0.21 | -0.08023 to 0.01228 | -0.34 | -0.05912 to 0.03339 | -0.15 |
| Adipocyte number per field | 4±1.826 | 4.25±1.708 | 15.25±8.221 | -10.04 to 9.544 | -0.07 | -21.04 to -1.456 | -0.69 | -20.79 to -1.206 | -0.68 |
| TRAP (n / mm^2) | 75.04±20.3 | 75.04±15.93 | 78.45±16.91 | -17.84 to 17.84 | 0.00 | -21.25 to 14.43 | -0.09 | -21.25 to 14.43 | -0.10 |
| OCN Positive area (%) | 28.22±8.799 | 25.2±5.127 | 18.03±5.505 | -4.005 to 10.05 | 0.21 | 3.164 to 17.22 | 0.57 | 0.1433 to 14.19 | 0.56 |
| P1NP (ng/ml) | 1.278±0.2616 | 1.107±0.2494 | 0.8687±0.2437 | -0.1464 to 0.4880 | 0.32 | 0.09230 to 0.7267 | 0.63 | -0.07850 to 0.5559 | 0.44 |
| CTX-1 mRNA level | 7.118±2.563 | 5.718±1.784 | 7.669±3.907 | -2.930 to 5.731 | 0.30 | -4.882 to 3.780 | -0.08 | -6.282 to 2.379 | -0.31 |
| TRAP mRNA level | 0.9478±0.09977 | 1.412±0.2007 | 1.351±0.3428 | -0.8188 to -0.1096 | -0.83 | -0.7578 to -0.04854 | -0.62 | -0.2936 to 0.4157 | 0.11 |
| CTSK mRNA level | 0.9136±0.09754 | 0.9039±0.09129 | 1.059±0.3684 | -0.3296 to 0.3490 | 0.05 | -0.4851 to 0.1935 | -0.26 | -0.4948 to 0.1837 | -0.28 |
| Colla1 mRNA level | 1.076±0.07934 | 2.212±0.9573 | 0.8048±0.1866 | -1.982 to -0.2880 | -0.64 | -0.5758 to 1.119 | 0.69 | 0.5595 to 2.254 | 0.71 |
| ALP mRNA level | 0.9344±0.1728 | 1.389±0.2768 | 0.7454±0.2905 | -0.8327 to -0.07614 | -0.70 | -0.1893 to 0.5673 | 0.37 | 0.2651 to 1.022 | 0.75 |
| Runx2 mRNA level | 1.19±0.06561 | 1.188±0.1175 | 0.8666±0.1455 | -0.1699 to 0.1733 | 0.01 | 0.1520 to 0.4952 | 0.82 | 0.1503 to 0.4935 | 0.77 |
| PPARy mRNA level | 0.8921±0.0855 | 1.156±0.1164 | 1.509±0.3136 | -0.5625 to 0.03534 | -0.79 | -0.9160 to -0.3181 | 0.31 | -0.6524 to -0.05456 | -0.60 |
| Adipoq mRNA level | 0.8657±0.1445 | 1.271±0.2741 | 1.825±0.1545 | -0.7054 to -0.1058 | -0.68 | -1.259 to -0.6594 | -0.95 | -0.8533 to -0.2538 | -0.78 |
| CD36 mRNA level | 0.9362±0.3835 | 2.126±0.3195 | 2.254±0.7317 | -1.957 to -0.4230 | -0.86 | -2.085 to -0.5507 | -0.71 | -0.8947 to 0.6392 | -0.11 |
| TGFβ mRNA level | 0.9849±0.0981 | 1.514±0.176 | 1.231±0.112 | -0.7283 to -0.3291 | -0.88 | -0.4459 to -0.04672 | -0.76 | 0.08278 to 0.4820 | 0.69 |
| BMSCs Collagen mRNA level | 1.95±0.446 | 1.658±0.4811 | 0.8583±0.2408 | -0.3127 to 0.8974 | 0.30 | 0.4868 to 1.697 | 0.84 | 0.1945 to 1.405 | 0.72 |
| BMSCs Runx2 mRNA level | 1.125±0.1988 | 1.597±0.508 | 0.8955±0.2807 | -1.003 to 0.05910 | -0.52 | -0.3014 to 0.7609 | 0.43 | 0.1706 to 1.233 | 0.65 |
| BMSCs PPARy mRNA level | 0.8026±0.1365 | 1.778±0.321 | 2.08±0.3961 | -1.432 to -0.5179 | -0.89 | -1.734 to -0.8199 | -0.91 | -0.7590 to 0.1549 | -0.39 |
| BMSCs CD36 mRNA level | 1.399±0.8371 | 3.535±1.225 | 6.732±0.9266 | -3.650 to -0.6213 | -0.71 | -6.847 to -3.819 | -0.95 | -4.712 to -1.683 | -0.83 |
| BMSCs TGFβ mRNA level | 0.9126±0.3966 | 2.769±0.7567 | 1.43±0.2786 | -2.634 to -1.078 | -0.84 | -1.295 to 0.2608 | -0.60 | 0.5607 to 2.117 | 0.76 |
| Runx2 protein level | 0.3571±0.04144 | 0.4161±0.1115 | 0.2156±0.02549 | -0.2350 to 0.1170 | -0.33 | -0.03452 to 0.3175 | 0.90 | 0.02451 to 0.3765 | 0.78 |
| PPARy protein level | 0.7718±0.4075 | 0.914±0.2042 | 1.076±0.1101 | -0.8204 to 0.5359 | -0.22 | -0.9825 to 0.3739 | -0.45 | -0.8402 to 0.5161 | -0.44 |
| Wnt5a mRNA level | 1.313±0.2072 | 1.415±0.4434 | 0.579±0.1918 | -0.5573 to 0.3529 | 0.44 | 0.2791 to 1.189 | 0.88 | 0.3812 to 1.291 | 0.77 |
| Wnt3a mRNA level | 0.893±0.06321 | 1.753±0.2516 | 0.8788±0.2198 | -1.154 to -0.5653 | -0.92 | -0.2802 to 0.3086 | 0.04 | 0.5795 to 1.168 | 0.88 |
| β-catenin mRNA level | 1.263±0.252 | 1.383±0.1901 | 0.7548±0.4966 | -0.6295 to 0.3895 | -0.26 | -0.0009724 to 1.018 | 0.54 | 0.1190 to 1.138 | 0.64 |
| LRP6 mRNA level | 1.252±0.2654 | 1.43±0.256 | 1.223±0.2376 | -0.5580 to 0.2016 | -0.32 | -0.3513 to 0.4083 | 0.06 | -0.1731 to 0.5865 | 0.39 |
| Tcf12 mRNA level | 1.191±0.1924 | 1.298±0.2784 | 1.071±0.2422 | -0.4670 to 0.2536 | -0.22 | -0.2403 to 0.4803 | 0.26 | -0.1336 to 0.5870 | 0.40 |
| Tcf7 mRNA level | 1.14±0.2397 | 1.52±0.3879 | 1.02±0.2174 | -0.8179 to 0.05689 | -0.51 | -0.3179 to 0.5569 | 0.25 | 0.06261 to 0.9374 | 0.62 |
| Wnt5a protein level | 0.4547±0.01057 | 0.4456±0.05398 | 0.2316±0.01401 | -0.07307 to 0.09112 | 0.12 | 0.1410 to 0.3052 | 0.99 | 0.1319 to 0.2961 | 0.94 |
| Wnt3a protein level | 0.5942±0.09493 | 0.5624±0.08841 | 0.2719±0.07366 | -0.1840 to 0.2476 | 0.17 | 0.1065 to 0.5381 | 0.88 | 0.07475 to 0.5063 | 0.87 |
| β-catenin protein level | 0.2481±0.04145 | 0.2152±0.01416 | 0.1234±0.02755 | -0.04187 to 0.1078 | 0.01 | 0.04991 to 0.1996 | 0.87 | 0.01693 to 0.1666 | 0.79 |
| Serum TC (mmol/L) | 2.747±0.2447 | 2.852±1.003 | 8.396±0.9402 | -1.314 to 1.104 | 1.00 | -6.858 to -4.440 | -0.97 | -6.753 to -4.335 | -0.94 |
| Serum TG (mmol/L) | 0.8659±0.2985 | 0.552±0.2496 | 0.6846±0.2506 | -0.08685 to 0.7146 | 0.50 | -0.2194 to 0.5821 | 0.31 | -0.5333 to 0.2682 | -0.26 |
| Serum LDL (mmol/L) | 0.4736±0.1283 | 0.5609±0.5207 | 3.415±0.8045 | -0.9244 to 0.7499 | -0.11 | -3.779 to -2.104 | -0.93 | -3.691 to -2.017 | -0.90 |
| Serum HDL (mmol/L) | 1.106±0.2666 | 1.278±0.4254 | 2.281±0.2951 | -0.6768 to 0.3317 | -0.24 | -1.679 to -0.6706 | -0.90 | -1.506 to -0.4980 | -0.81 |
| Liver TC (mmol/L) | 0.3646±0.1831 | 0.562±0.03442 | 0.5777±0.109 | -0.3843 to -0.01059 | -0.60 | -0.4000 to -0.02624 | -0.58 | -0.2025 to 0.1712 | -0.10 |
| Liver TG (mmol/L) | 0.6355±0.3257 | 0.7228±0.04243 | 1.16±0.3338 | -0.4927 to 0.3182 | -0.18 | -0.9296 to -0.1188 | -0.62 | -0.8424 to -0.03156 | -0.68 |
| RBG (mg/dl) | 155.7±14.48 | 142.2±14.76 | 153±28.8 | -17.19 to 44.19 | 0.42 | -27.99 to 33.39 | 0.06 | -41.49 to 19.89 | -0.23 |
| FBG (mg/dl) | 110.4±13.69 | 132.9±16.04 | 161.4±20.61 | -48.03 to 3.028 | -0.60 | -76.53 to -25.47 | -0.82 | -54.03 to -2.972 | -0.61 |

**Supplementary Table 2|** Means±SD, 95% CI, effect sizes (ES) for all data.
